# Pressure‐Driven Two‐Input 3D Microfluidic Logic Gates

**DOI:** 10.1002/advs.201903027

**Published:** 2019-12-17

**Authors:** Nazek El‐Atab, Javier Chavarrio Canas, Muhammad M. Hussain

**Affiliations:** ^1^ mmh Labs Electrical Engineering Computer Electrical Mathematical Science and Engineering Division King Abdullah University of Science and Technology (KAUST) Thuwal 23955‐6900 Saudi Arabia; ^2^ Electrical Engineering and Computer Science University of California Berkeley CA 94720‐1770 USA

**Keywords:** CO_2_ lasers, fluid mixing, fluid transport, logic gates, microfluidics

## Abstract

Microfluidics is a continuously growing field with potential not only in the fields of medical, chemical, and bioanalysis, but also in the domains of optics and information technology. Here, a pressure‐driven 3D microfluidic chip is demonstrated with multiple logic Boolean functions. The presence and absence of fluid at the output of the gates represent the binary signals 1 and 0, respectively. Therefore, the logic gates do not require a specially functionalized liquid to operate. The chip is based on a multilevel of poly(methyl methacrylate) (PMMA)‐based polymeric sheets with aligned microchannels while a flexible polyimide‐based sheet with a cantilever‐like structure is embedded to enable a one‐directional flow of the liquid. Several Boolean logic functions are realized (AND, OR, and XOR) using different fluids in addition to a half adder digital microfluidic circuit. The outputs of the logic gates are designed to be at different heights within the 3D chip to enable different pressure drops. The results show that the logic gates are operational for a specific range of flow rates, which is dependent on the microchannel dimensions, surface roughness, and fluid viscosity and therefore on their hydraulic resistance. The demonstrated approach enables simple cascading of logic gates for large‐scale microfluidic computing systems.

## Graphical abstract

Pressure‐driven 3D multilevel microfluidic logic gates are demonstrated. The logic gates operation is based on the fluid pressure within the microchannel, as a result, they can operate using any liquid without the need for a specially functionalized fluid. Multiple Boolean operations are realized with the capability of cascading them for large‐scale computations.

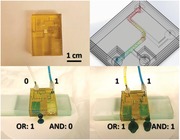

Recent developments in microfluidics^1^ are tackling technological challenges in a wide range of applications including chemistry,^2^ 3D printing,^3^ tissue engineering,^4^ drugs development,^5^ biomedical research,^6^ and most lately organs‐on‐chip.^7^, ^8^ Particularly, microfluidic devices which enable the handling of exceptionally low volumes of fluids in the range of micro‐ to pico‐liters are employed in biological and chemical analysis applications due to the precise manipulation of particles and liquids in a microscopic environment.^9^ Additionally, microfluidic devices can provide the opportunity to analyze, isolate, concentrate, control, and identify biomolecules with an improved sensitivity and throughput, in addition to being simpler and easier than conventional techniques. For instance, microfluidics have recently shown the capability to use very small amounts of samples and chemicals to detect cancer cells and their interaction with myeloid cells with high sensitivity, high resolution, fast analysis, and low cost.^10^

The future generation of microfluidic devices should be capable of performing in situ complex sample analysis and treatment. Today, integrated circuits can execute complex operations using electronic building blocks known as logic gates. This allows the system to autonomously make decisions following Boolean rules, thus eliminating the necessity for any manual intervention. Therefore, in order to automatically categorize, tag, isolate, and identify markers in complex fluidic samples such as blood, drugs, sweat, and so on, it is essential to include microfluidic logic functions in the miniaturized analysis. To date, multiple techniques have been demonstrated for logic computing including fluid flow resistance,^11^ electrochemical reactions,^12^ fluorescent molecular devices,^13^ nonlinearity in fluid viscosity,^14^ pneumatic pressure,^15^ and bubbles flowing in microchannels.^16^ Nonetheless, the major drawbacks of these approaches lie in the different interpretations of the input/output signals in addition to requirement for specially functionalized liquids for the logic gates to operate which makes the scaling and integration of multiple logic gates more complicated and challenging to achieve. As a result, microdroplet‐based microfluidic computation has received a growing attention in the past years due to its simple interpretation of output signals, where the presence and absence of the droplet represents the binary signals 1 and 0, respectively. However, this approach still requires the generation of microdroplets and their dispersal in another continuous liquid, in addition to the different required mechanisms for microdroplet movement such as relative flow resistance,^17^ applied voltage,^18^ magnetic field,^19^ etc.

To overcome these challenges, here, we report pressure‐driven 3D microfluidic logic gates that can operate using any fluid. The operation of the chip is based on the fluid pressure within the microchannels which is dictated by the flow rate defined using a syringe pump, a conventional tool that is used in most of the microfluidics applications. It is worth to note that the flow pressure within the channels depends on the hydraulic resistance as well which is a function of the microchannel dimensions, surface roughness, and fluid viscosity. However, these variables are usually fixed and known for a specific device and fluid. The 3D microfluidic chip is fabricated using CO_2_ ablation of poly(methyl methacrylate) (PMMA) sheets and their following bonding using a thermocompression process. It is important to note that PMMA has been one of the most popular material for microfluidic devices due to its transparency, low cost, rigidity, reliability, and compatibility with different existing biomolecular techniques.^20^ Therefore, this work enables easy integration of logic gates within most of the existing microfluidic devices. Several Boolean logic gates including AND, OR, and XOR are realized, in addition to a half adder digital microfluidic circuit, which is an essential component in the arithmetic logic unit (ALU) in microprocessors. In the demonstrated devices, the presence of the fluid at the output is interpreted as a binary signal 1 while the absence of the fluid is binary signal 0. The presented approach allows easy integration and cascading of microfluidic logic gates for complex logic computations.

The microfluidic chip is based on the 3D integration and stacking of four layers of PMMA sheets with microchannels. CO_2_ laser ablation with different power and speed recipes is used to create microchannels with different dimensions. The obtained shape of the microchannels is Gaussian due to their small widths (**Figure** **1**a–c) which restricts the proper development of the well, while macrochannels with large widths (>1 mm) result in a rectangular shape. In particular, due to this effect, changing the width of the microchannel while fixing the power and speed of the laser result in channels with different depths (Figure S1, Supporting Information). Moreover, for a specific fixed width, it is known that in order to get deeper channels, the power can be increased with a constant cutting speed or the speed of the laser can be reduced for a giver power. This ensures that the laser ablation effect on a specific area is more pronounced, as a result, a deeper channel is obtained. However, it is observed that the obtained width of the microchannel is also affected as shown in Figure 1a–c where a fixed power of 10% and a speed of 8% result in a channel width of 473 µm while reducing the speed to 4% results in a channel width of 518 µm. The same applies to the case when the laser speed is fixed and its power is modified, the width of the channel gets affected in addition to the depth. Therefore, in order to maintain the same width of the microchannel but to increase/reduce its depth, the laser recipe needs to be optimized in terms of both laser power and speed. Another observed effect during the CO_2_ laser ablation of the PMMA is that the surface roughness of the channel sidewalls increases at higher speeds as shown in Figure 1d–i. The surface roughness of the microchannel affects its hydraulic resistance and consequently the flow rate range of operation of the microfluidic logic gates.

**Figure 1:**
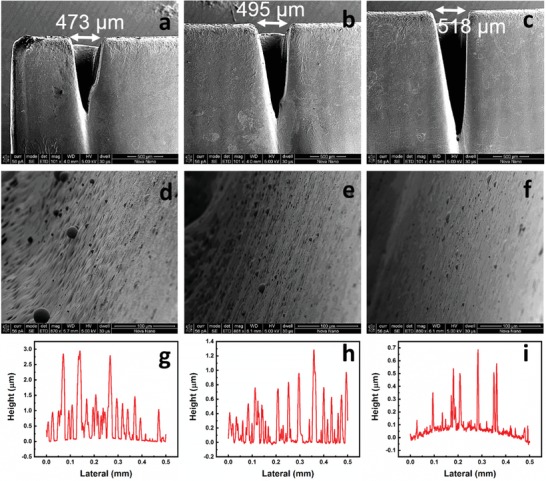
Microchannels fabrication in PMMA sheets and optimization using CO_2_ laser. Cross‐sectional scanning electron microscopy (SEM) image of the microchannel fabricated using a) a power of 10% and speed of 8%, b) a power of 10% and speed of 6%, c) a power of 10% and speed of 4%. The width of the microchannel is shown to be dependent on the laser speed. SEM image of the sidewall of the microchannel d) of (a), e) of (b), and f) of (c). Surface roughness using a profilometer of the sidewall of the microchannel g) of (a), h) of (b), and i) of (c).

Once the laser ablation recipes are optimized to get microchannels with different depths and widths, the PMMA sheets were then bonded using a thermocompression process as shown in **Figure** **2**a (Figure S2, Supporting Information). The microfluidic gates are pressure driven, therefore, the outputs of the two gates are designed to be located at different heights where the AND output is placed at a higher level than the OR output (Figure 2b). As a result, a higher pressure drop is required in order to activate the AND gate. This is achieved when fluid flows in both inputs *A* and *B* resulting in fluid flowing out of both AND and OR gates (*A*.*B* = 1, *A* + *B* = 1). However, when only one input is activated (*A*′.*B* = 1 or *A*.*B*′ = 1), the pressure drop across the AND gate is not large enough to turn it on and the fluid flows only out of the OR gate. One challenge that has been observed during the operation of the microfluidic chip with one fluid input (*A*′.*B* = 1 or *A*.*B*′ = 1) is the backflow of a small amount of the fluid into the other input due to capillary forces as shown in Figure 2c. To overcome this challenge, a flexible polyimide (PI)‐based cantilever‐like structure is embedded in between the PMMA sheets at the intersection of both inputs to enable a one‐directional flow of liquids as shown in Figure 2d. Using this approach, the AND and OR gates with two inputs are shown to work properly (Figure 2e–i, Videos S1–S3, Supporting Information). Blue and yellow colored water are used at inputs and the output is green colored which confirms the mixing of both inputs (Figure 2g–i). Similarly, an XOR gate is prepared using four layers of PMMA sheets and two PI layers with cantilever‐like structures to enable unidirectional flow of fluid between different PMMA levels and within the same level as well. The XOR gate is then combined with an AND gate to achieve a half adder microfluidic circuit (**Figure** **3**a,b). The operation of the half adder circuit is shown in Figure 3c–e (Videos S4–S6, Supporting Information) where the outputs in this case are the sum (sum = XOR (*A*,*B*) = *A*⊕*B*) and carry (carry = AND (*A*,*B*) = *A*.*B*).

**Figure 2:**
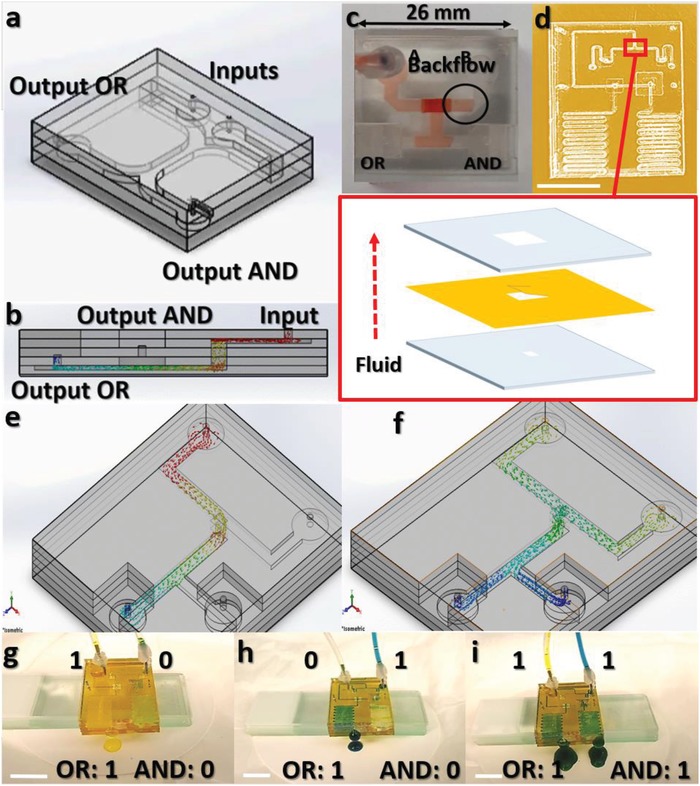
Microfluidic chip fabrication and design optimization. a) 3D microfluidic chip with multilevels of PMMA sheets. Microchannels are fabricated using CO_2_ laser followed by thermocompression of the different layers. b) Cross‐sectional illustration showing the fluid flow in the microchannels. c) Fabricated microfluidic chip showing a challenge of fluid backflow into the inputs. d) Optimized microfluidic chip design with scaled down microchannels and a flexible polyimide sheet embedded between the PMMA sheets to enable one‐directional flow of fluid. Inset shows the stack of the rigid PMMA and flexible polyimide sheets for fluid backflow blockade. Scale bar: 1 cm. Simulated 3D microfluidic chip showing the AND/OR outputs for e) *A* = 1 and *B* = 0, and f) *A* = 1 and *B* = 1. g) Optical image of the operation of the microfluidic chip with inputs *A* = 1 and *B* = 0. Scale bar: 1 cm. h) Optical image of the operation of the microfluidic chip with inputs *A* = 0 and *B* = 1. Scale bar: 1 cm. i) Optical image of the operation of the microfluidic chip with inputs *A* = 1 and *B* = 1. Scale bar: 1 cm.

**Figure 3:**
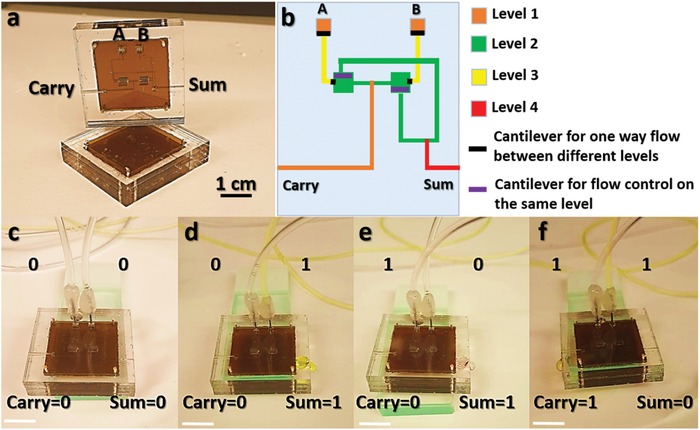
Half adder digital microfluidic circuit fabrication and design optimization. a) 3D half adder microfluidic circuit with four levels of PMMA sheets and two PI layers for unidirectional fluid flow. b) Half adder circuit design showing a color map where level 1 corresponds to the top PMMA sheet while level 4 corresponds to the bottom PMMA sheet. c) Optical image of the operation of the microfluidic half adder circuit with inputs *A* = 0 and *B* = 0. Scale bar: 1 cm. d) Optical image of the operation of the microfluidic half adder circuit with inputs *A* = 0 and *B* = 1. Scale bar: 1 cm. e) Optical image of the operation of the microfluidic half adder circuit with inputs *A* = 1 and *B* = 0. Scale bar: 1 cm. f) Optical image of the operation of the microfluidic half adder circuit with inputs *A* = 1 and *B* = 1. Scale bar: 1 cm.

To study the effect of scaling down the microchannels on the performance of the logic gates, different recipes were optimized to obtain either different depths, lengths, or widths while fixing the other dimensions as shown in **Figure** **4**a. For the depth study, the length and width of the microchannels are fixed at 38 mm and ≈300 µm, respectively. For the length study, the depth and width are fixed at ≈470 and ≈300 µm, respectively, while for the width study, the length and depth are fixed at 38 mm and ≈250 µm, respectively. The pressure drop between an input and an output in the chip is Δ*P* = *R*_H_ × *Q*, where *R*_H_ is the hydraulic resistance and *Q* is the flow rate. The hydraulic resistance is related to the dimensions of the channel and fluid according to *R*_H_ = $\;\frac{C_{\text{geometrical}}\times \mu \;\;\;\times L}{W\times D^{3}}$, where *L*, *W*, and *D* are the length, width and depth of the microchannel, respectively, *µ* is the viscosity of the fluid and *C*_geometrical_ is a geometrical factor that depends on the shape of the channel and its roughness.^21^ Therefore, since *R*_H_ is not simple to calculate especially with several variables that are not fixed for the whole channel such as the roughness and geometrical factor, the effect of the flow rate on the device performance is studied. In fact, to insert the fluids into inputs *A* and *B*, a syringe pump is used which enables the user to set the flow rate.

**Figure 4:**
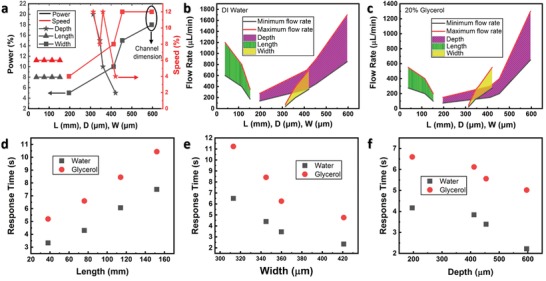
Operation of the microfluidic chip with different dimensions and fluids. a) CO_2_ laser recipes used for the fabrication of the different microchannels. For the depth study, the length and width are fixed at 38 mm and ≈300 µm, respectively. For the length study, the depth and width are fixed at ≈470 and ≈300 µm, respectively. For the width study, the length and depth are fixed at 38 mm and ≈250 µm, respectively. b) Operation regions of the microfluidic chip with different dimensions and DI water. c) Operation regions of the microfluidic chip with 20% glycerol and 80% DI water. Response time of the OR Boolean function for different d) lengths, e) widths, f) depths of the microchannels. The flow rate is fixed at 300 µL min^−1^ in the experiments.

Two fluids are tested: deionized (DI) water and 20:80 glycerol:water which results in a higher viscosity.^22^ It is observed that the logic gates are operational for a specific range of flow rates (Figure 4b,c). Using flow rates beyond the upper limit, one fluidic input (*A*′.*B* = 1 or *A*.*B*′ = 1) results in fluidic output in both AND and OR gates due to the high resulting fluid pressure. While using a flow rate below the lower limit results in no output from the AND gate when both inputs are turned on due to the very low pressure drop across the channel. In particular, the upper and lower flow rate limits are shown to reduce at higher lengths, smaller widths, and smaller depths. In fact, since the hydraulic resistance is directly proportional to the length and inversely proportional to the width and to the cubic depth, this will cause the increase of the hydraulic resistance and therefore increase the pressure drop across the channel. As a result, with one fluidic input, both AND and OR outputs are turned on. Therefore, as the length (depth, width) is increased (reduced, reduced), the hydraulic resistance is increased which require the reduction of the flow rate to maintain the same operational pressure drop (Figure 4b). Moreover, using a more viscous fluid (glycerol:water), the upper and lower limits of the operational flow rate are reduced compared to the water case (Figure 4c) due to the fact that the hydraulic resistance increases with the viscosity.

The surface roughness of the microfluidic channel plays an important role as well in determining the operational flow rate. To explain this effect, two devices with different dimensions are considered: Device 1 is chosen from the width study in Figure 4b, using *W* = 313 µm (while *D* and *L* are fixed at 250 µm and 38 mm, respectively), and Device 2 is chosen from the depth study, using *D* = 200 µm (while *W* and *L* are fixed at 300 µm and 38 mm, respectively). It can be concluded based on the dimensions of the microchannels in the two devices that Device 1 has a smaller *R*_H_ (due to the larger *W* and *D*), however, the upper and lower limits of the operational flow rates are smaller for Device 1 which is contradictory to the above explained reasoning. However, based on Figure 4a, it can be seen that the used recipe to create the microchannel in Device 1 shows a higher power and higher speed (*P* = 20%, *S* = 12%) than the recipe used for Device 2 speed (*P* = 5%, *S* = 4%). As a result, the surface roughness in Device 1 is higher which overcompensates the effect of the larger *D* and *W* and causes an overall increase in hydraulic resistance compared to the case of Device 2. As a result, the operational flow rate in Device 1 is lower than in Device 2.

When comparing the slope of the operational flow rate for the devices with different dimensions (Figure 4b,c), it can be observed that the depth study shows the smallest slope, the reason is the dependence of the *R*_H_ on 1/D^3^. However, it is also observed that the width study shows a reduction in operational flow rate by a larger factor than the depth study. Since the hydraulic resistance is inversely proportional to the width and directly proportional to the length, it is expected that the operational flow rate of both studies should change by around the same factor when a different fluid is inserted. However, our devices are based on the laser ablation of channels followed by the 3D stacking of PMMA sheets, as a result, when the width of the microchannel is increased, the fluid will be in contact with a larger surface area of polished PMMA (top of the channel). Therefore, in this case, as the width is increased, multiple opposing mechanisms that affect the hydraulic resistance compete including 1) increase in polished surface area in contact with the fluid (top of the channel), 2) increase in amount of fluid that is not in direct contact with the sidewalls of the channel (center of the channel), and 3) increase in surface roughness of the sidewalls (due to the higher laser power and speed as shown in Figure 4a). As a result, the overall hydraulic resistance experienced by the fluid is increased by a smaller factor when the width is reduced than in the case when the length is increased. Finally, the response time for the fluidic OR gate is studied for the devices with different dimensions and different fluids using a fixed flow rate of 300 µL min^−1^ (Figure 4d–f). The results show that the response time increases linearly with length (Figure 4d) but not with width and depth (Figure 4e,f). This can be explained by the fact that the hydraulic resistance in the latter cases is affected by the change in the surface roughness as well due to the different optimized recipes for the width and depth studies (Figure 4a) in addition to the *R*_H_ variation with 1/D^3^.

In conclusion, a 3D multilevel microfluidic chip with different Boolean logic gates and a digital microfluidic circuit (AND, OR, XOR, and half adder) is demonstrated. The microfluidic logic gates operation is based on the pressure drop between the inputs and outputs of the device, which is a function of both the hydraulic resistance (fixed for a given device) and the flow rate set using a syringe pump. The presence of the fluid at the output represents a logic signal 1 while its absence is a logic signal 0. As a result, the demonstrated microfluidic logic devices can be easily used with any fluid and cascaded to achieve integrated and complex computations. The results show that the logic gates are operational for a specific range of flow rates which is dependent on the microchannel dimensions, surface roughness, and fluid viscosity. Finally, the response time of the logic gates is studied for devices with different dimensions, the results confirm its dependency on the channel hydraulic resistance.

## Experimental Section

*Fabrication of PMMA Sheets with Microchannels*: A CO_2_ laser tool (Universal Laser Systems) with maximum power of 75 W and speed of 223 mm s^−1^ was used to cut the PMMA sheets and create the microchannels. A power of 10% corresponds to 7.5 W while a speed of 10% corresponds to 22.3 mm s^−1^. PMMA sheets with 2 mm thickness were used in addition to a flexible 120‐µm‐thick PI sheet for blocking the backflow of fluids. The device consists of four PMMA sheets and an embedded PI sheet. The PI sheet was patterned using the CO_2_ laser to obtain a cantilever‐like structure to enable the one‐directional flow of fluid. All the layers were aligned using metallic pins inserted at the edges of the device. Food coloring was used to color the water at the inputs of the microfluidic logic gates.

*Bonding of the PMMA Sheets*: A thermocompression tool was used to bond the several layers of the device as shown in Figure S2 in the Supporting Information. The bonding procedure was the following: first, the PMMA sheets were aligned using metallic pins, next the microfluidic device was placed between two silicon wafers to avoid direct contact between the hot plates and the PMMA sheets. The complete sandwich of silicon wafers and PMMA sheets was then placed in between the hotplates in the thermocompression tool. The temperature of the top and bottom plates was set to 120 °C and the spacing between the plates was narrowed down to the exact thickness of the sandwich (no applied pressure) to provide heat transfer by conduction and avoid trapping air within the device which would otherwise result in air bubbles between the PMMA sheets. Once the temperature of the system reaches 120 °C (higher than the glass transition temperature of PMMA), a pressure of 20–40 lbs was applied between the hotplates to compress and bond the layers. The heaters were then turned off and the cooling valves were opened to cool down the device. The pressure was kept constant until the temperature reached a value of 90 °C. The bonding process is shown in Figure S2 in the Supporting Information.

*Characterization*: The microfluidic devices were tested using a Harvard syringe pump. Two syringes with 60 mL capacity were installed. Deionized water and 20:80 Glycerol:DI were used during the experiments. In the apparatus, the volumetric flow set point (flow rate) was changed to study its effect on the operation of the logic gates.

## Conflict of Interest

The authors declare no conflict of interest.

## Author Contributions

*N.E.‐A. conceptualized the idea. M.M.H. directed the study. N.E.‐A. designed the system, characterized the devices, and analyzed the data, J.C.C. assisted in the fabrication and characterization of the microfluidic logic gates, and all authors contributed to writing the manuscript*.

## Supporting information

Supporting Information

Supplemental Video 1

Supplemental Video 2

Supplemental Video 3

Supplemental Video 4

Supplemental Video 5

Supplemental Video 6
