# Supplementary material for: Pressure‐Driven Two‐Input 3D Microfluidic Logic Gates
Source: Adv Sci (Weinh). 2019 Dec 17;7(2):1903027. doi: 10.1002/advs.201903027 (PMC6974944; doi:10.1002/advs.201903027)
Supplement: Supplementary file 1 — Supporting Information [file ADVS-7-1903027-s001.pdf]

Copyright WILEY-VCH Verlag GmbH & Co. KGaA, 69469 Weinheim, Germany, 2019.

ADVANCED SCIENCE
Open Access

Supporting Information

for *Adv. Sci.,* DOI: 10.1002/advs.201903027

Pressure-Driven Two-Input 3D Microfluidic Logic Gates

*Nazek El-Atab, Javier Chavarrio Canas, and Muhammad M. Hussain**

Copyright WILEY-VCH Verlag GmbH & Co. KGaA, 69469 Weinheim, Germany, 2019.

Supporting Information

Pressure-Driven Two-Input 3D Microfluidic Logic Gates

*Nazek El-Atab, Javier Chavarrio Canas and Muhammad M. Hussain**

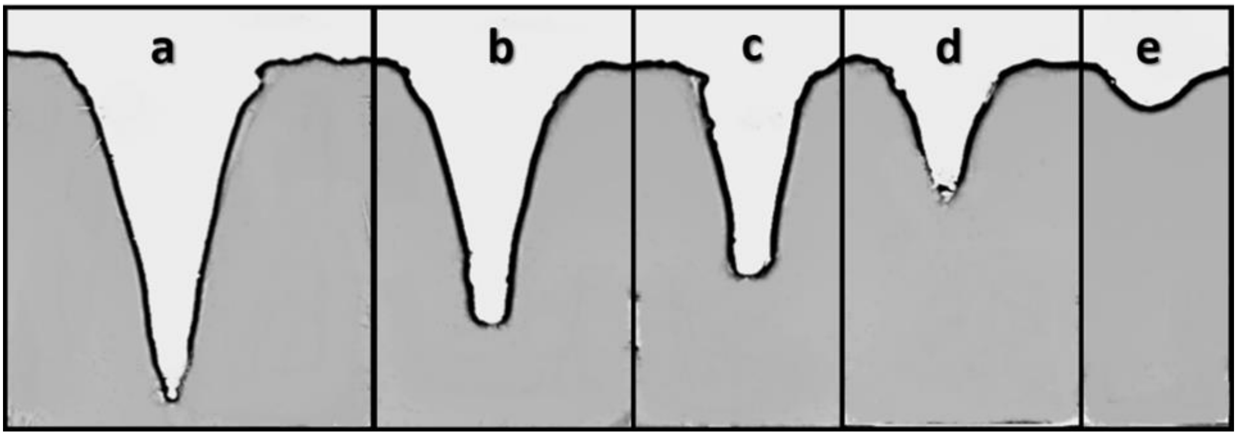

Figure S1. Optical images of the microchannels obtained by laser ablating PMMA sheets using the same laser power and speed (P = 20%, S = 6%) but for different widths a) 250 µm, b) 200 µm, c) 150 µm, d) 100 µm, e) 50 µm. The images show that for different widths, the depths of the channels is also changed.

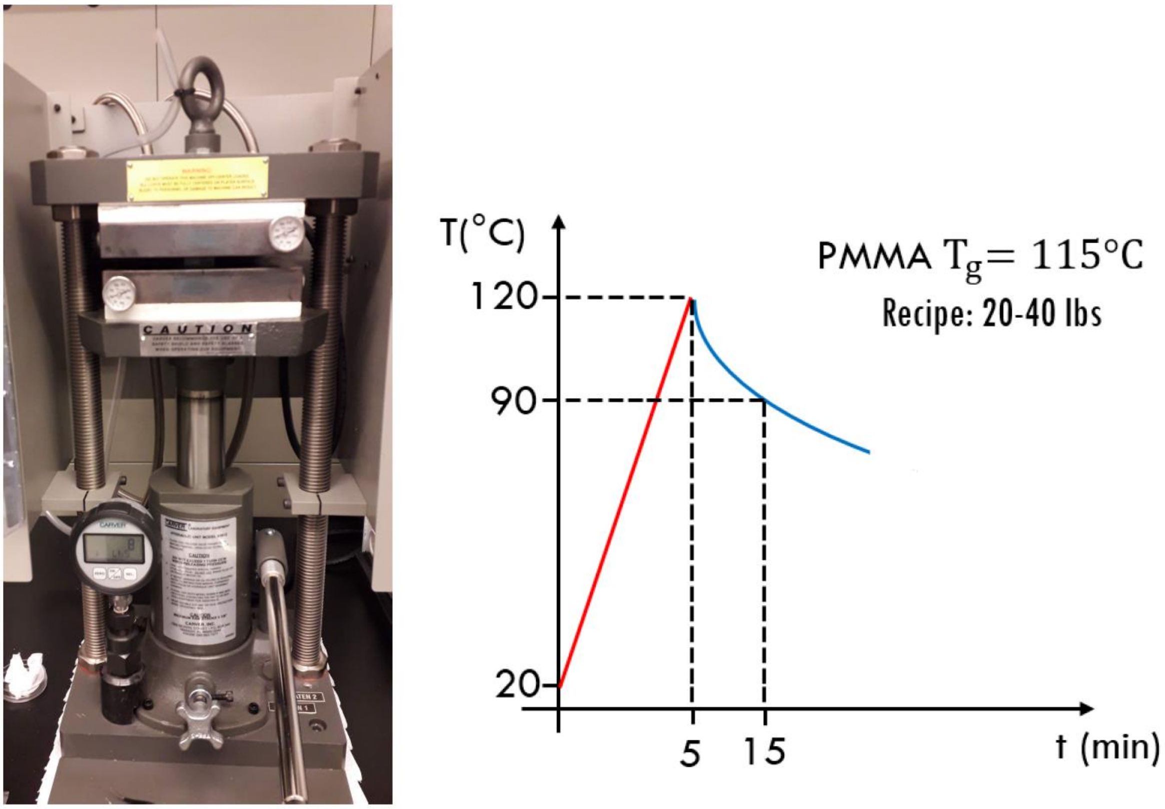

Figure S2. Thermo-compression tool used to bond the PMMA layers in the microfluidics device with the applied bonding recipe.

Video S1. Operation of the microfluidic logic gate with input AB'=1 where yellow colored water is inserted. The OR output is turned on while the AND output shows no fluidic output.

Video S2. Operation of the microfluidic logic gate with input A'B=1 where blue colored water is inserted. The OR output is turned on while the AND output shows no fluidic output.

Video S3. Operation of the microfluidic logic gate with input AB=1 where yellow and blue died water are inserted. The OR and AND outputs is turned on showing a green colored water which confirms the mixing of the two inputs (yellow and blue).

Video S4. Operation of the microfluidic half adder with input A'B=1 where yellow colored water is inserted. The sum output is turned on while the carry output shows no fluidic output.

Video S5. Operation of the microfluidic half adder with input AB’=1 where light-pink colored water is inserted. The sum output is turned on while the carry output shows no fluidic output.

Video S6. Operation of the microfluidic half adder with input AB=1 where yellow and light-pink died water are inserted. The carry output in this case is turned on showing fluidic output unlike the sum output.
